# Unique circulating cytokine profile of rheumatoid arthritis patients with nodules

**DOI:** 10.1186/1479-5876-9-S2-P48

**Published:** 2011-11-23

**Authors:** Bridget Hodkinson, Pieter WA Meyer, Eustatius Musenge, Mahmood Ally, Ronlad Anderson, Mohammed Tikly

**Affiliations:** 1 Chris Hani Baragwanath Hospital, University of the Witwatersrand, Johannesburg, South Africa; 2 Medical Research Council Unit for Inflammation and Immunity, University of Pretoria and NHLS, South Africa; 3 Biostatistics and Epidemiology Division, School of Public Health, University of the Witwatersrand, South Africa; 4 Dept. of Internal Medicine, University of Pretoria, South Africa

## Background

Immunohistochemical studies suggest that the rheumatoid nodule (RN) is a Th1 granuloma [1], with focal vasculitis [2], yet the pathogenesis remains unclear and little is known about circulating cytokines in these patients.

## Objective

We measured circulating cytokines of DMARD-naïve early RA patients, and compared profiles of patients with RN to those without RN.

## Methods

A cross-sectional cohort of 149 DMARD-naïve adults with early RA (symptom duration ≤ 2 years). Assessments included a smoking history, the presence of subcutaneous RN, the Simplified Disease Activity Index (SDAI), and X-rays scored using the modified Larsen method, Rheumatoid Factor (RF) and anti cyclic citrullinated peptides (aCCP) tests. Serum cytokines were measured using the Bio-Plex® suspension array system. Differences between patients with RN and those without RN were explored using univariate and multivariate techniques, correcting for SDAI by regression analysis.

## Results

Of 149 patients (120 females), the majority were Black Africans (93%) and 34 (22.8%) had RN. Patients with RN were more likely to be males, but neither smoking history nor symptom duration was significantly different between the two groups. RN patients had severe RA with higher SDAI (p=0.04) and X-ray scores (p=0.004), and higher RF (p=0.005) and aCCP (p=0.04) titers. Cytokine profiles in the RN patients showed significant elevation of predominantly Th1 cytokines, with higher interferon-gamma (IFN-G) (p=0.05), interleukin (IL)-12 (p=0.02), and IL-2 (p=0.05). In addition, cytokines of macrophage (TNF-a, p=0.16) and fibroblast origin (IL-7, p=0.05 and vascular endothelial growth factor, p=0.03) were significantly higher in these patients. Multivariate analysis showed IL-7 (p<0.001) and IFN-G (p=0.001) to be independently significant.

## Conclusion

DMARD-naïve early RA patients with RN have a unique serum cytokine profile, with significant elevation of predominantly Th1 and fibroblast cytokines.
